# Editorial: Frontiers in Neuro Oncology and Neurosurgery

**DOI:** 10.3390/brainsci13040565

**Published:** 2023-03-28

**Authors:** Roberto Altieri

**Affiliations:** 1 Department of Neurological Surgery, Policlinico “G. Rodolico-S. Marco” University Hospital, Viale Carlo Azeglio CIampi, 1, 95121 Catania, Italy; roberto.altieri.87@gmail.com; 2 Interdisciplinary Research Center on Brain Tumors Diagnosis and Treatment, University of Catania, 95123 Catania, Italy

Despite advances in our knowledge and treatments, Central Nervous System (CNS) Tumors remain the most difficult clinical challenge for the worldwide medical community.

We recently acquired a new classification based on the molecular features of each CNS tumor with a better prognostic stratification, but we are still far from the solution of this terrible puzzle.

The crucial battles of every war in this field are disputed in the periphery and the peritumoral zones (PTZ), which have received special scientific interest in the last few years.

Our Editorial on the Special Issue entitled “Frontiers in Neuro Oncology and Neurosurgery” provides an overview about this neuro-oncological topic from a multidisciplinary point of view.

The standard of care for Glioma is: maximal safe resection and adjuvant concomitant radiotherapy and chemotherapy.

First, it is crucial to clarify what “maximal safe resection” means for setting the best onco-functional balance.

Prof. Berger reviews the possibility of a supramaximal resection for Glioblastoma (GBM) and discusses the evidence surrounding the composition of peri-tumoral FLAIR hyperintensity, outcomes associated with FLAIRectomy, future directions of the field, and potential implications for patients. It is a leader opinion and opens the way to changing daily clinical practice [1].

Spena et al. provide a critical review with a meta-analysis of this hot topic including 19 articles. They underline that there is no consensus about the amount of volume that must be resected beyond the Enhancing Nodule (EN) to have an Overall Survival (OS) gain. This study is methodologically flawless and critically discusses a crucial issue to find a common denominator concerning supratotal resection [2].

Prof. Duffau et al., instead, provide an overview on the PTZ in Diffuse Low-Grade Gliomas (DLGG). They state that a connectome-based “supratotal” surgical-resection-improved OS limits risk of malignant transformation while improving quality of life, thanks to better seizure control. Moreover, the post-operative medical treatments can be modulated according to the pattern of peritumoral infiltration. This paper represents the state of the art regarding the PTZ in DLGG, summarizing the experience of the greatest neuroscientists of this field [3].

Zeppa et al., in a retrospective single-center study, compared the results obtained using 5-ALA and sodium fluoresceine in GBM surgery, concluding that there is no difference in achieving a gross total resection using fluorochromes. This study provides an original and innovative point of view about intraoperative fluorescence use. Many papers describe the pros and cons of each fluorochrome, but little is known about the combination of 5-ALA and sodium fluorescein. The authors describe the results of their experience [4].

Bruno et al., moreover, in a detailed retrospective single-center study, evaluated the impact on OS of clinical and molecular features, extent of resection, adjuvant treatments, and treatment-related complications in 135 elderly patients affected by GBM. It is a complete and methodologically correct study investigating the whole topic without neglecting any details [5].

It is well demonstrated that GBM can recur in the surgical cavity. Lo Greco et al. reviewed the role of adjuvant radiotherapy, underlining the role of radiomics and PET/MRI imaging, stating that it has a relatively good outcome and avoids severe toxicity. This study represents a critical review of the literature completing the section about the treatment of PTZ beyond the EN [6].

The study of functional features of the brain surrounding a tumor is another issue of special interest.

Cargnelutti et al. evaluated the possibility of neuroplasticity and compensation in a retrospective study on patients affected by malignant brain tumors localized on the sensorimotor cortex. It is an original study of great interest for clinical/surgical purposes. Despite it being a retrospective paper on a small series, the completeness of data evaluated and the correctness of the scientific methodology provide good study strength [7].

Colpitts et al., in an original and interesting case report, suggest that the propagating waves of spreading depolarization may potentially play a role in both non-epileptic transient neurologic deficits and tumor progression in patients affected by Gliomas. Obviously, the scientific weakness of the paper is related to the intrinsic nature of the case reports, but the originality of the authors’ idea opens the way for further investigations [8].

Yang et al. discuss the mechanism of intraoperative stimulation-related epilepsy using DTI-based graph theoretical analysis and suggest a basis to predict it in presurgical planning. The Chinese group provide us with a good overview of a crucial issue [9].

From a pathological and biological point of view, different aspects of this topic are explored.

Broggi et al. provide a histological definition of the central core and “Flair hyperintensity region” beyond the EN in GBM, evaluating 109 samples collected with the aid of neuro-navigation and 5-ALA. They found that FLAIR areas consisted in: (I) fragments of white matter with focal or diffused areas of tumor cell infiltration in 76% of cases; (II) a mixture of white matter with reactive astrogliosis and grey matter with perineuronal satellitosis in 15%, and (III) tumor tissue in 9%. It is a prospective study that defines the PTZ from a pathological point of view. The strength of the methodology especially regards the sampling’s correctness with a multimodal intraoperative protocol (Neuronavigation, 5-ALA, etc.) [10].

Menna et al., instead, investigated the mechanisms of crosstalk in the tumoral microenvironment and, with their systematic review, confirmed that microglia play a paramount role in promoting GBM progression and relapse after treatment. This systematic review describes this complex issue by exploring every detail [11].

Concerning the “periphery”, Piazza et al. wrote an elegant and interesting paper demonstrating the correlation between the amount of circulating exosomal DNA, glioma volume, and mitotic activity; this is particularly noted with low-grade gliomas. These results suggest a possible role of exoDNAs in diagnosing brain glioma with a special clinical benefit for detecting early recurrent high-grade gliomas and asymptomatic low-grade gliomas [12].

Wang et al. studied the role of Tropomyosin 4 (TPM4) (an oncogenic gene known to be involved in many malignancies) in 998 glioma patients and suggest that TPM4 is significantly correlated with more malignant characteristics of gliomas, potentially through involvement in epithelial-to-mesenchymal transition with a worse prognosis. It is an original and elegant study on a great number of patients [13].

Regarding other intracranial tumors, Armocida et al., in a multicentric study, investigated the mortality rates, grading, characteristics, and outcomes of patients affected by giant meningiomas, while Wang et al. investigated the influence factors for predicting the Ki-67 index in patients with pituitary adenomas [14,15].

As Guest Editors for this Research Topic, we hope that you enjoy these extremely interesting manuscripts.
